# Intrinsic and Extrinsic Determinants of T Cell Metabolism in Health and Disease

**DOI:** 10.3389/fmolb.2019.00118

**Published:** 2019-10-25

**Authors:** Haydn Munford, Sarah Dimeloe

**Affiliations:** Institute of Immunology and Immunotherapy, Institute of Metabolism and Systems Research, College of Medical and Dental Sciences, University of Birmingham, Birmingham, United Kingdom

**Keywords:** T cell, metabolism, cancer, immunology, adaptive immune cells, glycolysis, mitochondria, hypoxia

## Abstract

T lymphocytes are a critical component of the adaptive immune system, with key roles in the immune response to infection and cancer. Their activity is fundamentally underpinned by dynamic, regulated changes in their metabolism. This ensures adequate availability of energy and biosynthetic precursors for clonal expansion and effector function, and also directly regulates cell signaling, gene transcription, and protein translation. In health, distinct T cells subtypes demonstrate differences in intrinsic metabolic capacity which correlate with their specialized immune functions. In disease, T cells with impaired immune function appear to be likewise metabolically impaired. Furthermore, diseased tissue environments—through inadequate provision of nutrients and oxygen, or accumulation of metabolic intermediates, end-products, and cytokines- can impose metabolic insufficiency upon these cells, and further compound intrinsic impairments. These intrinsic and extrinsic determinants of T cell metabolism and their potential compound effects, together with the mechanisms involved form the subject of this review. We will also discuss how dysfunctional metabolic pathways may be therapeutically targeted to restore normal T cell function in disease.

T lymphocytes are a critical component of the adaptive immune system. Two major subsets of T cells, CD4^+^, and CD8^+^ T cells act as co-ordinators and effectors of the immune response, respectively. Upon recognition of a specific pathogen- or tumor-derived antigen in the context of appropriate co-stimulatory signals, T cells clonally expand and traffic to tissues, where their effector functions include direct killing of infected and malignant cells, and secretion of cytokines to coordinate the activity of other immune cells. These significant changes in T cell activity are accompanied—and indeed fundamentally underpinned—by substantial changes in their metabolism. This so-called “metabolic reprogramming” of T cells ensures adequate availability of energy and biosynthetic precursors for clonal expansion and synthesis of effector molecules. Fascinatingly, these metabolic changes also directly regulate T cell signaling, gene transcription and protein translation. Since, in this manner, metabolism determines T cell activity, logically, subtypes of T cells with superior immune function (e.g., memory T cells) also demonstrate superior metabolic capacity. Conversely, it appears that T cells with impaired immune function (e.g., in the context of primary immune deficiency, chronic infection, immune senescence, and cancer) are likewise metabolically impaired. Furthermore, diseased tissue environments (e.g., the tumor microenvironment) may impose metabolic insufficiency upon these cells, and compound intrinsic impairments, through inadequate provision of nutrients and oxygen, or accumulation of metabolic intermediates and end-products (metabolites). These intrinsic and extrinsic determinants of T cell metabolism and their compound effects, together with the mechanisms involved form the subject of this review. We will also discuss how dysfunctional metabolic pathways may be therapeutically targeted to restore normal T cell function in disease.

## Metabolic Reprogramming and T Cell Function

The most significant change in T cell metabolism following antigen recognition is a shift toward an anabolic profile which supports cell division and synthesis of effector molecules. Previously metabolically quiescent cells—relying largely on oxidative phosphorylation (OXPHOS) of glucose and fatty acids in the mitochondria to generate ATP for homeostatic processes—begin to take up large amounts of glucose and amino acids, and alter their metabolism of these substrates, to meet their rapidly changing requirements. Increased activity of kinases including phosphoinositide 3-kinase (Pi3K), Akt, and mechanistic target of rapamycin (mTOR), together with transcription factors such as c-Myc, Foxo1, IRF4, and Nur77 drive these metabolic changes and are reviewed elsewhere in greater depth (Roy et al., 2018).

**Figure 1:**
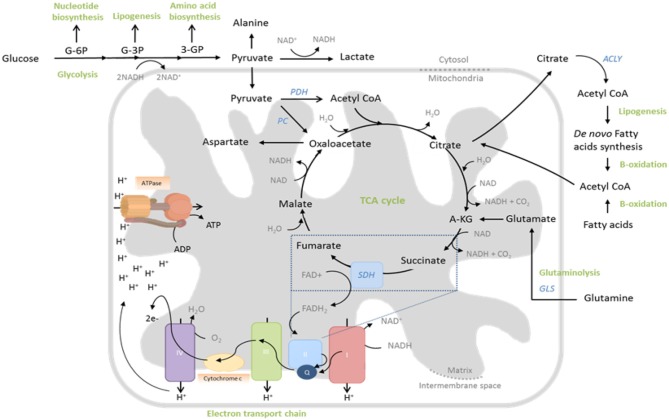
Central metabolic pathways whose activity is regulated to support T cell function. During glycolysis, glucose is converted to pyruvate through sequential enzymatic reactions occurring in the cytosol. Intermediates of this process can be further metabolized to yield precursors for synthesis of nucleic acids, lipids and amino acids. Pyruvate can either enter the mitochondria to drive the tricarboxylic acid (TCA) cycle or be reduced to lactate and excreted. In the TCA cycle, glucose-derived pyruvate, once converted to acetyl-CoA by pyruvate dehydrogenase (PDH), is sequentially oxidized to yield biosynthetic intermediates (e.g., citrate) and reduced electron carriers (NADH and FADH_2_) that drive oxidative phosphorylation (OXPHOS) by the electron transport chain (ETC). Fatty acids and glutamine can also fuel the TCA cycle, following fatty acid oxidation and glutaminolysis, respectively. The ETC consists of five multi-subunit complexes, which are located within the inner mitochondrial membrane. Complexes I and II accept electrons from reduced NADH and FADH_2_, respectively, and pass them, via Coenzyme Q (Q), to Complex III and subsequently via cytochrome c (C) to complex IV. Complex IV finally transfers the electrons to molecular oxygen as final electron acceptor to reduce oxygen to water. In parallel, protons (H^+^) are pumped across the mitochondrial inner membrane into the inter-membrane space, building up an electrochemical gradient across the mitochondrial inner membrane. This can then be used by Complex V (ATP-Synthase) to generate adenosine triphosphate (ATP) from adenosine diphosphate (ADP) and phosphate.

Glucose uptake is increased by trafficking of the Glut1 transporter to the cell surface (Jacobs et al., 2008), whereas amino acids, particularly glutamine, influx into the cell via heightened expression of transporters such as LAT1, SNAT-1, SNAT-2, and ASCT2 (Carr et al., 2010; Sinclair et al., 2013; Nakaya et al., 2014). Glucose is first metabolized to pyruvate in the cytoplasm (Figure 1), which also occurs (to a lesser extent) in quiescent cells. However, the fate of this pyruvate is altered following T cell activation. Whereas, resting cells convert most pyruvate to acetyl-CoA for subsequent mitochondrial oxidation, activated cells rather reduce substantial amounts of pyruvate to lactate, which is then excreted (Jacobs et al., 2008; Wang et al., 2011; Figure 1). This process, which is normally a metabolic adaptation of cells to low oxygen, occurs even in the presence of adequate oxygen and is therefore called “aerobic glycolysis.” Despite providing much less ATP per molecule of glucose than mitochondrial oxidation, aerobic glycolysis permits more rapid metabolism of influxed glucose to pyruvate (through rapid regeneration of the metabolic cofactor NAD^+^ and maintenance of favorable AMP/ATP ratios) thereby ensuring abundance of the many biosynthetic precursors generated as intermediates of this process (Figure 1), for nucleic acid, protein, and lipid biosynthesis—all critically required by cells which are rapidly proliferating and producing effector molecules. Furthermore, heightened glycolytic activity also directly promotes the expression of effector molecules, through regulatory effects on protein translation. Specifically, the glycolytic enzyme glyceraldehyde 3-phosphate dehydrogenase (GAPDH), when not actively engaged in glycolysis binds certain mRNAs [e.g., of the cytokine interferon-gamma (IFN-γ)] via AU-rich regions in their 3′ UTR, preventing translation; a mechanism which is disengaged in highly-glycolytic, activated T cells (Chang et al., 2013).

In parallel to this significant increase in aerobic glycolysis in activated T cells, there is also a substantial (albeit lesser) increase in mitochondrial glucose oxidation (Sena et al., 2013; Blagih et al., 2015). Here, glucose-derived pyruvate enters the mitochondria and is converted into acetyl-CoA, which is sequentially oxidized in the tricarboxylic acid (TCA) cycle. This produces carbon dioxide and water, but also yields GTP and reduces the electron carriers NADH and FADH_2_, which, together, drive the mitochondrial electron transport chain (ETC) and production of ATP and reactive oxygen species (ROS) (Figure 1). The TCA cycle oxidizes not only glucose in this manner, but also fatty acids and glutamine, which enter as acetyl-CoA and α-ketoglutarate, respectively (Figure 1). Indeed glutamine, like glucose, is also critically required by proliferating T cells (Wang et al., 2011) and as well as promoting mTOR activity (Nakaya et al., 2014), it also significantly contributes to the TCA cycle, especially when glucose availability is limiting (Blagih et al., 2015). Consistently, as well as increasing expression of glutamine transporters, activated T cells also upregulate glutaminolytic enzymes that metabolize glutamine to α-ketoglutarate (Wang et al., 2011; Figure 1). Mechanistically, increased mitochondrial OXPHOS of these substrates is linked to T cell proliferation and function, not only through generation of required ATP, but also of ROS that stabilize transcription factors, promoting effector molecule expression (Sena et al., 2013; Okoye et al., 2015) and supporting the activity of metabolic pathways that maintain redox homeostasis, such as the malate-aspartate shuttle (Bailis et al., 2019). Furthermore, TCA cycle intermediates are used for anabolic processes, as well as post-translational modifications of proteins—notably the acetylation of histones, permitting gene transcription (Peng et al., 2016; Bailis et al., 2019; Qiu et al., 2019) and enzymes such as GAPDH, thereby augmenting their function (Balmer et al., 2016). Mitochondria also sustain calcium signaling during T cell activation, by translocating to the immune synapse where (relative to their activity and membrane potential) they buffer incoming calcium, preventing calcium-dependent channel closure (Schwindling et al., 2010).

## T Cell-intrinsic Determinants of Metabolism and Function

Given how, in this manner, metabolism is intimately linked to T cell function, it is not surprising that T cell subsets with different immune functional capacity also demonstrate different intrinsic metabolic capacity. This concept was initially delineated by comparing antigen-inexperienced “naïve” T cells with “memory” T cells which remain following resolution of an immune response and protect the host against a secondary antigen encounter due to their specialist functions. Other studies have additionally found that subsets of helper CD4^+^ T cells, for example pro-inflammatory, effector Th1, Th2, and Th17 cells are metabolically distinct from regulatory CD4^+^ T cells which suppress and resolve immune responses. Memory T cells demonstrate significantly larger, more complex mitochondria than naïve cells, with increased respiratory capacity, particularly for fatty acids (van der Windt et al., 2012, 2013; Gubser et al., 2013; Buck et al., 2016; Dimeloe et al., 2016). This was linked to increased survival and motility under hypoxia *in vitro* (mimicking peripheral tissue environments) (Dimeloe et al., 2014, 2016) and also to greater longevity *in vivo* (van der Windt et al., 2012). Additionally, greater mitochondrial capacity was implicated in the rapid proliferation and cytokine production of memory cells, through provision of ATP to support rapid induction of glycolysis (van der Windt et al., 2013). Memory T cells are moreover better equipped for this rapid adoption of glycolysis, by abundant glycolytic enzyme expression in their cytoplasm (Gubser et al., 2013; Dimeloe et al., 2016). Other metabolic adaptations of memory T cells—and particularly those which reside in the tissues—include heightened capacity for the uptake, synthesis, storage, and breakdown of lipids (O'Sullivan et al., 2014; Cui et al., 2015; Pan et al., 2017). For example, critical to long-term memory T cell survival is expression of the glycerol transporter AQP9, which mediates uptake of glycerol for triglyceride synthesis and storage (Cui et al., 2015). Additionally, memory T cells demonstrate abundant mitochondria-endoplasmic reticulum contact sites, serving as “immunometabolic hubs,” where key signaling proteins, ion channels, and metabolic enzymes interact at the subcellular level to bring about rapid changes in metabolism upon antigen encounter (Bantug et al., 2018a). Within the CD4^+^ T cell compartment, it has been reported that inflammatory Th1 and Th17 cells are highly glycolytic, whereas regulatory T cells (TReg) can tolerate low glucose availability but appear to rely on fatty acid oxidation for their suppressive function (Dumitru et al., 2018).

By contrast, whilst certain T cell subsets are metabolically primed for their optimal immune function, in other contexts it appears that T cell functional impairment is accompanied by intrinsic metabolic insufficiency. For example, a number of primary immune deficiencies (PIDs) are caused by underlying systemic metabolic defects, for example in nucleic or amino acid synthesis pathways, which manifest as immune dysfunction, since rapidly proliferating lymphocytes are particularly affected. These disorders are extensively reviewed elsewhere (Parvaneh et al., 2014; Fischer, 2015). Other PIDs are associated with hereditary defects in the signaling pathways that instruct T cell metabolic reprograming, leading to dysfunctional metabolic and functional T cell phenotypes. One example is the gain—of—function mutations in Pi3K, which cause increased AKT phosphorylation, hyperactivation of mTOR and consequent sustained high glucose uptake. Somewhat counterintuitively, this enforced “hyper-metabolic” state is actually associated with loss of effector T cell function and increased susceptibility to infection, but is similar to the metabolic phenotype of an exhausted or senescent T cell (see below) (Lucas et al., 2014; Bantug et al., 2018b). Another example is hereditary deficiency of the complement receptor CD46, which is associated with increased susceptibility to intracellular infection and abortive T cell activation. Mechanistically, it was found that T cell CD46 ligation by complement C3b instructs increased expression of Glut1 and the large neutral amino acid transporter LAT1, resulting in increased uptake of glucose, leucine, and phenylalanine. In parallel, CD46 signaling promotes activation of mTOR by these same amino acids, through upregulation of intracellular amino acid sensing machinery (Kolev et al., 2015).

**Figure 2:**
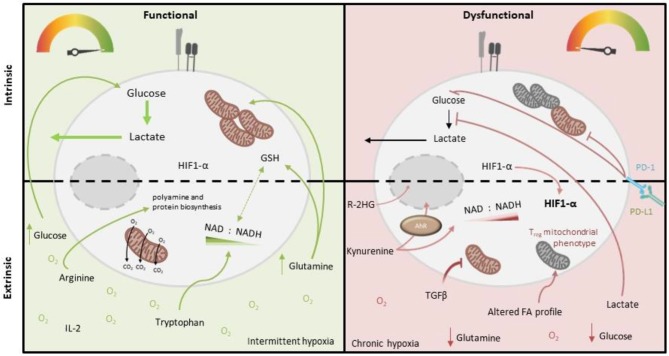
Intrinsic and extrinsic metabolic factors affecting function of tumor-infiltrating T cells. Tumor-infiltrating T cells (right), compared to more functional T cells from other locations (left), demonstrate variable capacity for glucose uptake and metabolism, and decreased mitochondrial mass and function. PD-1 signaling and deficiency of PGC1-α are both implicated in these cell-intrinsic metabolic impairments. Extrinsic factors which may also impose metabolic insufficiency on tumor-infiltrating T cells include reduced abundance of glucose, glutamine (which is both an important oxidative substrate and regulates redox balance via GSH and NAD/H), arginine (which critically supports polyamine and protein biosynthesis) and tryptophan (which is a precursor for NAD synthesis), as well as accumulated lactate (which inhibits glycolysis), kynurenine (which is immunosuppressive) and other metabolites, specific to the tumor type (e.g., 2-HG, which impairs T cell transcription factor activity). Altered oxygen abundance and fatty acid profiles as well as an altered cytokine milieu (i.e., abundance of IL-2/IL-7 vs. TGF-β) also play a role, for example TGF-β inhibits T cell mitochondrial function.

T cell-intrinsic metabolic impairment is also reported in the context of chronic viral infections, both in murine models and in human patient cohorts. In a mouse model of chronic viral infection, metabolic changes in viral-specific T cells were already observed in the early stages of infection (compared to those with an acute resolving infection), such as decreased rates of aerobic glycolysis and reduced mitochondrial respiratory capacity (Bengsch et al., 2016). This was associated with a significant loss of mitochondrial membrane potential (mitochondrial depolarization), which undermines mitochondrial efficiency whilst also causing heightened ROS production. These metabolic impairments persisted in the T cells in established chronic viral infection, which were also functionally impaired (Bengsch et al., 2016). An interesting human correlate of these findings was reported in the context of chronic infection with hepatitis B virus (HBV). Virus-specific T cells were analyzed from individuals chronically co-infected with both HBV and cytomegalovirus (CMV). Compared to the more functional CMV-specific T cells, poorly-functional HBV-specific cells demonstrated high Glut1 surface expression and capacity for glucose uptake, but significantly lower mitochondrial membrane potential. Consistently, they were much more dependent upon glucose availability for cytokine production than CMV-specific cells. Interestingly in this case, both the mitochondrial and functional impairment of HBV-specific cells could be rescued *in vitro* by culture with the cytokine IL-12 (Schurich et al., 2016). A more recent human study of HBV-specific T cells in chronic viral infection—in this case compared with those specific for influenza—also found significant mitochondrial abnormalities. These could be corrected by *in vitro* treatment with mitochondrial anti-oxidants, which also improved immune function (Fisicaro et al., 2017). Mechanistically, in the murine model, signaling via the co-inhibitory “checkpoint” receptor PD-1 was implicated, since the metabolic abnormalities were not present in viral-specific T cells lacking PD-1. Indeed, PD-1 signaling is reported to directly alter T cell mitochondrial morphology and function, as well as inhibit glycolysis (Patsoukis et al., 2015; Ogando et al., 2019; Figure 2), which is consistent with it antagonizing co-stimulatory CD28 signaling via Pi3K/Akt. Additionally, a role for peroxisome proliferator-activated receptor γ (PPARγ) co-activator 1α (PGC-1α), a key transcriptional regulator of genes controlling mitochondrial biogenesis was identified, since both metabolic and functional impairments were prevented by artificially overexpressing PGC-1α (Bengsch et al., 2016).

These two molecules have also been linked to T cell metabolic and functional exhaustion in cancer (Figure 2), both in experimental cancer models and human disease. A study of human tumor infiltrating CD8^+^ T lymphocytes (CD8^+^ TILs) in non-small cell lung cancer examined the transcriptional and metabolic profile of these cells relative to their level of expression of PD-1. The study found that “PD-1 high” cells had distinctive metabolic features compared to those with intermediate or low PD-1 expression, namely heightened capacity for both glucose and lipid uptake, but also depolarized mitochondria with altered morphology and reduced numbers of cristae. This metabolic phenotype was associated with impaired production of key anti-tumor cytokines compared to cells expressing lower levels of PD-1 (Thommen et al., 2018). In a murine melanoma model, tumor-infiltrating CD8^+^ T cells were found to have suppressed mitochondrial function and mass when compared to those from the draining lymph node, which was induced upon entry into the tumor microenvironment and associated with decreased expression of PGC-1α (Scharping et al., 2016; Figure 2). Notably in this study, overexpression of PGC-1α rescued intratumoural T cell metabolism and improved immune function.

Other intrinsic mechanisms of metabolic and functional T cell exhaustion have also been proposed in cancer. For example, a recent study has reported that CD8^+^ TILs in murine and human melanoma demonstrate impaired activity of the glycolytic enzyme Enolase-1, despite high expression of both mRNA and protein, indicating a potential post-translational modification. This was associated with reduced effector functionality of these cells, which could be partially restored by provision of pyruvate, which also augmented both glycolytic and mitochondrial activity of these cells (Gemta et al., 2019).

A similar but slightly different phenomenon to T cell exhaustion is the accumulation of terminally differentiated, senescent T cells, which also occurs in the context of chronic viral infection and cancer, and also as a part of human aging. T cell senescence is characterized by telomere shortening due to the loss of telomerase expression, which results in impaired cellular proliferation. Unlike exhausted T cells, senescent T cells are considered to be capable of producing cytokines, and do not always demonstrate increased levels of co-inhibitory receptors. They are instead defined by their characteristic surface expression of a number of other molecules, for example CD57 and KLRG1. Additionally, they can be defined by their re-expression of the naïve T cell-associated protein CD45RA, or by decreased expression of the co-stimulatory receptors CD27 and CD28 (Henson et al., 2014). In a study of the metabolic capacity of human T cell populations, senescent CD8^+^ T cells—in this case defined by surface expression of CD45RA and absence of CD27—were found to have significantly fewer mitochondria than more functional memory T cell populations, which were furthermore largely depolarized. Consistently, these cells also demonstrated substantial levels of mitochondrial and total cellular ROS. These senescent cells also demonstrated low Glut1 surface expression, however their basal glycolytic rate was comparable to memory T cells (Henson et al., 2014). In this study, the mitochondrial defects of senescent T cells could be partially rescued by treatment *in vitro* with an inhibitor of p38 MAP kinase signaling, which also increased telomerase activity and proliferative capacity of these cells (Henson et al., 2014). A subsequent study has directly implicated mitochondrial dysfunction in the development of T cell senescence, finding a direct correlation between mitochondrial ROS abundance and telomere length in human CD8^+^ T cells and furthermore demonstrating that treatment of these cells with mitochondrial anti-oxidants prevented telomere shortening *in vitro* (Sanderson and Simon, 2017). More recently, another human study, investigating highly cytotoxic CD8^+^CD28^−^ T cells, which also expand with age and are implicated in inflammatory disease, found these cells to be highly glycolytic. This work mechanistically implicated decreased expression of the NAD^+^-dependent protein deacetylase SIRT1, and consequent increased abundance of the transcription factor Foxo1, since overexpression of Foxo1 in CD28-sufficient T cells also increased glycolytic capacity and cytotoxicity (Jeng et al., 2018).

## T Cell-extrinsic Determinants of Metabolism and Function

In addition to these T cell intrinsic factors, aspects of their environment can also impair their metabolic activity and competence, thereby impacting immune function. These factors have been best characterized to date in the tumor microenvironment and include availability of glucose, lipids, and other nutrients, abundance of metabolic intermediates and end-products, the cytokine milieu and importantly oxygen tensions, which are discussed in turn below.

## Glucose Availability

Similarly to active immune cells, cancer cells also typically consume glucose at high rates, while also producing increased lactate. This metabolic phenotype results in a glucose-deprived microenvironment, which is reported to encourage an exhausted-like state with diminished anti-tumor activity in T cells (Chang et al., 2015; Ho et al., 2015; Figure 2). Accordingly, tumor expression of Glut1 correlates with a lower infiltration of CD8^+^ T cells in tumors (Ottensmeier et al., 2016) and poor prognosis in pancreatic adenocarcinoma (Davis-Yadley et al., 2016). Mechanistically, glucose restriction results in reduced glycolytic intermediates such as phosphoenolpyruvate (PEP) which can impair calcium sarco/endoplasmic reticulum Ca^2+^-ATPase channel regulation, resulting in defective NFAT signaling (Ho et al., 2015). Moreover, IFN-γ is particularly sensitive to the metabolic state of T cells. As described above, GAPDH plays a regulatory role in IFN-γ translation, while glucose-derived acetyl-CoA promotes the acetylation of *IFNG*-associated histones. Both of these are therefore crucial for effector T cell function (Chang et al., 2013; Peng et al., 2016). The importance of glucose uptake has also been demonstrated in T cell responses in ovarian carcinoma (OvCa). Song et al. found that ascitic fluid attained from OvCa patients could inhibit glucose uptake, which mechanistically triggered N-linked protein glycosylation defects in T cells, leading to IRE1α/XBP1-driven suppression of mitochondrial activity and ultimately impaired IFN-γ production (Song et al., 2018). Interestingly it was recently reported that exogenous acetate may be able to compensate in glucose-limiting conditions to maintain T cell effector function, owing its importance to histone acetylation (Qiu et al., 2019). This reveals potential therapeutic windows for future research. Conversely to effector T cells, regulatory T cells are reported to have adaptations that enable a selective metabolic advantage in low glucose environments, which may promote their induction within TMEs (Angelin et al., 2017).

## Lipids

Fatty acid (FA) homeostasis is perturbed in several cancers (Rohrig and Schulze, 2016; Tirinato et al., 2017). Given the variable dependence of FA oxidation vs. FA synthesis among T cell subsets, alterations in FA availability may impact each subset differently (Figure 2). For example, memory T cells require exogenous FA in culture medium in order to develop appropriately (Rolf et al., 2013) and therefore their growth may be altered within a tumor microenvironment. It has recently been shown that TILs have increased PPARα expression when compared to non-tumor residing T cells. PPARα is a nuclear receptor able to induce the expression of Cpt1a, required for the oxidation of lipids. This may therefore allow TILs to utilize FAs from the TME (Zhang et al., 2017). FAs have also been shown, at least in the gut, to induce regulatory T cell differentiation, which may therefore also occur in the tumor microenvironment. Mechanistically, this could be a result of altered G protein-coupled receptor-mediated signaling and thus independent of lipid metabolism, which is yet to be fully determined (Furusawa et al., 2013).

## Amino Acids

T cells require a number of other nutrients in addition to glucose to maintain their proliferation, survival, and effector function in sub-optimal environments. Although not an essential amino acid, exogenous glutamine is required for proper mTORC1 function, as well as maintaining TCA metabolism via α-ketoglutarate anaplerosis, as described above. The importance of glutamine metabolism has been highlighted during the activation and expansion of CD4^+^ T cells, where they become sensitized to glutaminase inhibition under both normoxia and hypoxia (Sener et al., 2016). In an interesting recent study however the importance of exogenous glutamine for T cell function was investigated. Nabe et al. reported how the restriction of glutamine metabolism during TCR-mediated activation counterintuitively led to reduced T-cell exhaustion, while enhancing the antitumor activity of tumor-specific CD8+ T cells (Nabe et al., 2018). Despite its importance, relatively few studies have as yet investigated the alterations of glutamine abundance and metabolism in the context of disease.

T cells are also reliant on arginine (which is a precursor for polyamine and protein biosynthesis) and tryptophan (required for proliferation), which have both been implicated in impaired T cell responses to either viral infection, cancer or both (Mbongue et al., 2015). Chronic HBV and HIV-1 viral infection both result in reduced arginine and tryptophan concentrations, which strongly decrease anti-viral T cell responses (Qin et al., 2013; Pallett et al., 2015). Myeloid-derived precursor cells (MDSC) can accumulate in both viral infection and cancer. Via increased arginase (ARG) expression, MDSCs can result in reduced arginine concentrations, contributing to impaired T cell proliferation (Pallett et al., 2015; Rodriguez et al., 2017; Figure 2). The exact mechanisms of how arginine is sensed within T cells has not been fully elucidated, however the involvement of, BAZ1B, PSIP1, and Translin are thought to promote T cell survival via arginine recognition (Geiger et al., 2016). Further studies by Geiger et al. revealed that CD8^+^ T cells supplemented with L-Arginine had a higher survival capacity both *in vitro* and *in vivo*, compared to arginine deplete controls. Furthermore, these CD8^+^ T cells sustained a central memory T cell phenotype and produced larger quantities of IFN-γ upon re-stimulation (Geiger et al., 2016). To further highlight the importance of arginine in anti-tumor T cell responses, there is a negative correlation between ARG expression and T cell abundance in lung adenocarcinoma (LUAD) and lung squamous cell carcinoma (Miret et al., 2019).

In certain tumor microenvironments the tryptophan metabolizing enzyme IDO is increased, leading to reduced tryptophan and a subsequent increase in kynurenine (Kyn) concentrations (Lob et al., 2009; Wainwright et al., 2012; Figure 2). In relation to this, expression levels of another tryptophan-catabolizing enzyme, TDO, correlate with poor clinical outcomes in patients with triple-negative breast cancer (Greene et al., 2019). Although decreased tryptophan can impact T cell proliferation, it may be the immunosuppressive role of Kyn that is more important in determining T cell effector functions. Kynurenine is able to bind the aryl hydrocarbon transcription (AhR) factor complex to stimulate CD4^+^ T cell differentiation. In particular, AhR signaling is able to dictate the differentiation of activated CD4^+^ T cells to regulatory T cells (Mezrich et al., 2010). More recently, it has been shown that AhR signaling may also drive PD-1 expression in CD8^+^ T cells both in tumor-bearing mice and patients. The same group also identified the transporters SLC7A8 and PAT4 as being essential for Kyn uptake (Liu et al., 2018).

Although not a focus of this review, the depletion of other amino acids, metabolites and ions may also be important to T cell responses. For example, mice on a serine and glycine-restricted diet have impaired antigen-driven CD8^+^ effector T cell expansion and pathogen clearance (Ma et al., 2017). A recent publication by Vodnala et al. pinpoint potassium as being important in global nutrient insufficiencies in T cells (Vodnala et al., 2019). They demonstrate a potassium-induced state of starvation through reduced nutrient uptake and consumption. This may become of particular interest in anti-tumor T cell responses, where local potassium concentration in the TME can exceed 40 mM (Baixauli et al., 2019).

## Lactate and Other Metabolites

In addition to Kyn as described above, other metabolic intermediates and end-products accumulate in diseased tissue and may impact T cell function, for example lactate (Figure 2). Effector T cells require the regeneration of NAD^+^ to sustain GAPDH activity and glycolysis. Even at very low glucose concentrations T cells are able to fuel proliferation; however the influx of lactate and subsequent reversal of lactate dehydrogenase (LDH) reduces their ability to regenerate NAD and thus maintain glycolysis (Angelin et al., 2017). Both CD4^+^ and CD8^+^ T cells have the ability to import lactate via differential transporter activities, which acts to reduced glycolytic flux and migration (Haas et al., 2015). Furthermore, lactate is able to induce high IL-17 production and loss of cytolytic activity in CD4^+^ and CD8^+^ T cells, respectively. In support of this, Fischer et al. report that lactic acid completely abolishes IL-2 and IFN-γ production in CTLs, in a pH independent manner (Fischer et al., 2007). Additionally, a lactate-rich environment has been shown to reduce both the Th1 pool and their ability to produce IFN-γ, as well as a concomitant increase in the percentage of regulatory T cells (Comito et al., 2019). A relatively recent study by Xia et al. show a novel role of lactic-acid in the induction of apoptosis in naïve T cells which they attribute to a loss in FAK family-interacting protein of 200 kDa (Xia et al., 2017). Again regulatory T cells appear to be unaffected by this metabolic imbalance, owing to Foxp3-dependent reprograming of T cell metabolism suppressing Myc and glycolysis, enhancing oxidative phosphorylation, and increasing NAD oxidation (Angelin et al., 2017). Ultimately, Foxp3 expression allows the resistance of regulatory T cells to the suppressive effects of lactate and may explain how regulatory T cells promote cancer evasion from effector T cells in the TME. It is therefore unsurprising that lactate metabolism in T cells also directly correlates with survival in patients with colorectal cancer. Roseweir et al. observed that the expression of monocarboxylate transporter (MCT) 1, MCT2, and lactate dehydrogenase (LDH) 1 and LDH5, were inversely correlated with survival (Roseweir et al., 2019). Encouragingly, inhibition of LDH via overexpression of miR-34a can restore effector T proliferation and increase IFN-γ and GZMB expression (Ping et al., 2018), highlighting a crucial role of LDH in T cell function.

Another metabolite which accumulates in certain tumors is (R)-2-hydroxyglutarate (R-2-HG). This is produced by tumors harboring isocitrate dehydrogenase (IDH) mutations. R-2-HG accumulates within the TME and is taken up by T cells, resulting in suppression of T cell activity. Mechanistically R-2-HG–mediated T-cell inhibition is thought to reduce NFAT target expression via modulation of T-cell receptor signaling and AMPK signaling (Bunse et al., 2018).

## The Cytokine Milieu

The impact of different cytokines and their receptor signaling pathways on T cell metabolism is yet to be fully explored in depth. However, some studies have highlighted the potential for cytokines to both positively or negatively regulate aspects of T cell metabolism, or that of other immune cell types. For example, IL-2 and IL-7 appear to strongly promote glycolysis (Wofford et al., 2008; Chang et al., 2013; van der Windt et al., 2013; Preston et al., 2015) whereas IL-15 drives mitochondrial biogenesis (van der Windt et al., 2012, 2013). Interesting recent studies identified that TGF-β can directly suppress both glycolysis and mitochondrial oxidative function of natural killer cells (Zaiatz-Bittencourt et al., 2018) and effector memory CD4^+^ T cells (Dimeloe et al., 2019), which may be relevant in a tumor microenvironment where TGF-β can be abundant. It appears therefore that the balance of different cytokines present in a T cell's immediate environment may also determine metabolic capacity and function (Figure 2).

## Hypoxia

T cells encounter wide ranging oxygen tensions throughout their life span. For instance, developing thymocytes are subjected to reasonably low oxygen concentrations in the thymus, while mature cells within the circulation encounter far higher oxygen tensions (Ohta et al., 2011). Tumor microenvironments and chronically inflamed tissues are often hypoxic, with reported oxygen levels averaging about 1–2% in solid tumors (Muz et al., 2015; Figure 2). Exposure to hypoxic environments induces stabilization of HIF-1α which drives the metabolic adaptations required to survive and function there. However, in T cells HIF-1α expression is also concomitant with activation, irrespective of oxygen concentrations (pseudo hypoxia) (Tao et al., 2015). This feature of pseudo hypoxia is therefore an important part of T cell development although HIF-1α expression may also be beneficial in oxygen depleted niches. In terms of the overall effects of hypoxia on T cell function, Gropper et al. showed that CD8^+^ T cells are able to survive and mature in hypoxic conditions, but have a slowed proliferation rate. They further demonstrated the increased production of Granzyme-B, which enhanced CD8^+^ T cell cytolytic potential (Gropper et al., 2017). More specifically, HIF-1α activation polarizes CD8^+^ T cells to favor glycolysis, which is obligatory for their effector function (Finlay et al., 2012). This was further shown to encourage the formation of tumoricidal memory populations (Sukumar et al., 2013). Furthermore, the importance of HIF-1α signaling for an anti-tumor response has recently been shown by Palazon et al. who demonstrated how HIF-1α knockout CD8^+^ T cells are unable to infiltrate and kill subcutaneously grafted melanoma cells (Palazon et al., 2017). Contrary to these reports of HIF-1α promoting CD8^+^ T cell function, an independent study reported that partial reductions in HIF-1α actually enhanced cytokine production in activated CD8^+^ T cells (Guo et al., 2009), suggesting a fine balance of HIF-1α expression might be required for appropriate function. Aside from the induction and role of HIF-1α, other studies indicate that hypoxia may have additional immunosuppressive effects, based on observations that the expression of co-inhibitors, such as CTLA-4, LAG-3, and CD244 are increased on T cells under low oxygen tensions (Doedens et al., 2013). Moreover, hypoxic tumors tend to accumulate adenosine within the TME, which can negatively regulate both the T cell activation and effector function required for a successful anti-tumor response (Kobie et al., 2006; Deaglio et al., 2007). Therefore, it appears that HIF-1α plays a natural role in T cell development, while hypoxia may be detrimental to effector function, depending on the degree and persistence of hypoxia. More work will be needed to fully elucidate the role of hypoxia in T cell responses.

## Compound Effects and Therapeutic Perspective

It is very likely that in certain disease contexts, for example the solid tumor microenvironment, that T cells with cell-intrinsic metabolic defects as described above, encounter an unfavorable metabolic environment which further compounds their overall metabolic and functional impairment. For example we could predict that mitochondrial dysfunction may be worsened by exposure to cytokines such as TGF-β or hypoxia, or that T cells with reduced capacity for glucose uptake, or greater dependency on glycolysis vs. mitochondrial OXPHOS would be particularly susceptible to glucose limitation and lactate abundance. Whilst this hypothesis remains to be fully confirmed by experimental *in vivo* studies and analyses of tumor-derived T cell populations from patients, it would seem prudent when designing novel therapeutic approaches to target both T cell-intrinsic and—extrinsic metabolic and functional factors. Indeed, as examples of this approach, interesting recent studies using experimental models of melanoma identified that anti-PD-1 treatment was most effective when tumor oxidative metabolism was also genetically or pharmacologically inhibited—resulting in higher intra-tumoral oxygen levels and improved T cell anti-tumor function (Scharping et al., 2017; Najjar et al., 2019). Analyses of samples from patients with melanoma provided an interesting translational correlate of these findings, demonstrating that following treatment with anti-PD-1, significantly lower numbers of functional, cytokine-producing CD8^+^ T cells in were found in tumors with the highest oxygen consumption rates (Najjar et al., 2019). Another approach taken by the same research group was to provide 4-1BB co-stimulation alongside PD-1 blockade, which led to increased CD8^+^ T cell mitochondrial capacity and a favorable antitumor response, again in an experimental melanoma model (Menk et al., 2018). Alternatively, through the use of pharmacologic compounds, Chamato et al. showed that enhanced ROS can strongly activate mitochondrial function, leading to improved anti-tumor responses (Chamoto et al., 2017), again dependent on synergized PD-1 blockade.

## Author Contributions

HM and SD contributed to writing and reviewing the manuscript.

### Conflict of Interest

The authors declare that the research was conducted in the absence of any commercial or financial relationships that could be construed as a potential conflict of interest.
